# Metagenomic Analysis of Bacterial Communities of Antarctic Surface Snow

**DOI:** 10.3389/fmicb.2016.00398

**Published:** 2016-03-31

**Authors:** Anna Lopatina, Sofia Medvedeva, Sergey Shmakov, Maria D. Logacheva, Vjacheslav Krylenkov, Konstantin Severinov

**Affiliations:** ^1^Department of Molecular Genetics of Cell, Institute of Molecular Genetics, Russian Academy of SciencesMoscow, Russia; ^2^Department of Molecular Genetics of Microorganisms, Institute of Gene Biology, Russian Academy of SciencesMoscow, Russia; ^3^Research Complex of “Nanobiotechnology”, Saint-Petersburg State Polytechnical UniversitySaint-Petersburg, Russia; ^4^Center for Data-Intensive Biomedicine and Biotechnology, Skolkovo Institute of Science and TechnologySkolkovo, Russia; ^5^Belozersky Institute of Physico-Chemical Biology, Moscow State UniversityMoscow, Russia; ^6^Department of Botany, Saint-Petersburg State UniversitySaint-Petersburg, Russia

**Keywords:** CRISPR, Antarctica, microbial diversity, genetics, metagenomics

## Abstract

The diversity of bacteria present in surface snow around four Russian stations in Eastern Antarctica was studied by high throughput sequencing of amplified 16S rRNA gene fragments and shotgun metagenomic sequencing. Considerable class- and genus-level variation between the samples was revealed indicating a presence of inter-site diversity of bacteria in Antarctic snow. *Flavobacterium* was a major genus in one sampling site and was also detected in other sites. The diversity of flavobacterial type II-C CRISPR spacers in the samples was investigated by metagenome sequencing. Thousands of unique spacers were revealed with less than 35% overlap between the sampling sites, indicating an enormous natural variety of flavobacterial CRISPR spacers and, by extension, high level of adaptive activity of the corresponding CRISPR-Cas system. None of the spacers matched known spacers of flavobacterial isolates from the Northern hemisphere. Moreover, the percentage of spacers with matches with Antarctic metagenomic sequences obtained in this work was significantly higher than with sequences from much larger publically available environmental metagenomic database. The results indicate that despite the overall very high level of diversity, Antarctic Flavobacteria comprise a separate pool that experiences pressures from mobile genetic elements different from those present in other parts of the world. The results also establish analysis of metagenomic CRISPR spacer content as a powerful tool to study bacterial populations diversity.

## Introduction

Snow covers about 35% of the Earth's surface—permanently or for varying times during the year—and is thus a major climatic and ecological system (Miteva, 2008). It directly affects climate, moisture budget and sea level, and also serves as an interface between different ecosystems (Pomeroy and Brun, 2001; Davis et al., 2005; Zhang, 2005; Hinkler et al., 2008). Snow ecosystems are characterized by harsh conditions such as low temperatures, low atmospheric humidity, low liquid water availability, and high levels of radiation (Cowan and Tow, 2004). The amount of microorganisms on the surface snow varies from 10^2^ cells per milliliter of melted snow on South Pole (Carpenter et al., 2000) to 10^2^–10^5^ in high mountain and Arctic snow (Segawa et al., 2005; Amato et al., 2007; Liu et al., 2009; Harding et al., 2011). Bacterial diversity from Arctic and alpine snow has been intensively investigated during the last few decades (Blank et al., 2002; Bachy et al., 2011; Varin et al., 2012; Hell et al., 2013; Maccario et al., 2014). Bacteria of several phylogenetic groups have been detected; most were of *Alphaproteobacteria, Betaproteobacteria, Gammaproteobacteria, Firmicutes, Bacteroidetes*, and *Actinobacteria* classes (Segawa et al., 2005; Amato et al., 2007; Møller et al., 2013; Maccario et al., 2014; Cameron et al., 2015). Recently, a metagenomic study of Arctic spring snow suggested that snow bacteria can be adapted to photochemical reactions and oxidative stress in addition to cold stress (Maccario et al., 2014), and therefore may form specific communities.

Microorganisms on the surface snow in Antarctica were also analyzed (Carpenter et al., 2000; Brinkmeyer et al., 2003; Christner et al., 2003; Fujii et al., 2010; Lopatina et al., 2013). Representatives of *Proteobacteria, Bacteroidetes, Cyanobacteria*, and *Verrucomicrobia* have been detected in different sampling sites (Brinkmeyer et al., 2003; Lopatina et al., 2013). Antarctic snow microbial communities have been found to be metabolically active based on the measurements of radioactive thymidine and leucine incorporation (Carpenter et al., 2000; Lopatina et al., 2013). Microbial activity on the surface snow of Dome C was also suggested by the presence of exopolysaccharide-like debris on the DAPI-stained filters and by scanning electron microscopy (Michaud et al., 2014). Also, evidence of active microbial life in the coastal snow of Antarctica was gained during analysis of “red snow” bacterial composition, which was dominated by green alga, producing pigment astaxanthin (Fujii et al., 2010).

Comparative metagenomic analysis of Antarctic show has not been undertaken so far. Availability of such data, particularly from multiple sampling sites, could reveal the presence of particular snow-specific communities or, conversely, point to introduction of snow microorganisms through eolian effects. Here, we performed amplicon library and metagenomic analysis of bacterial sequences from Antarctic snow collected around four Russian stations in Eastern Antarctica. The results reveal very considerable variation between the sites and show clear evidence of deposition of marine bacteria in stations close to open water. We also performed metagenomic analysis of CRISPR spacers in a *Flavobacterium* common in Antarctic snow. The results revealed, surprisingly, a staggering diversity of CRISPR spacers that is distinct from the limited known diversity of flavobacterial spacers from the Northern hemisphere, suggesting that diversity of flavobacterial CRISPR spacers is generated and maintained locally in response to specific genetic parasites.

## Methods

### Study sites

**Figure 1:**
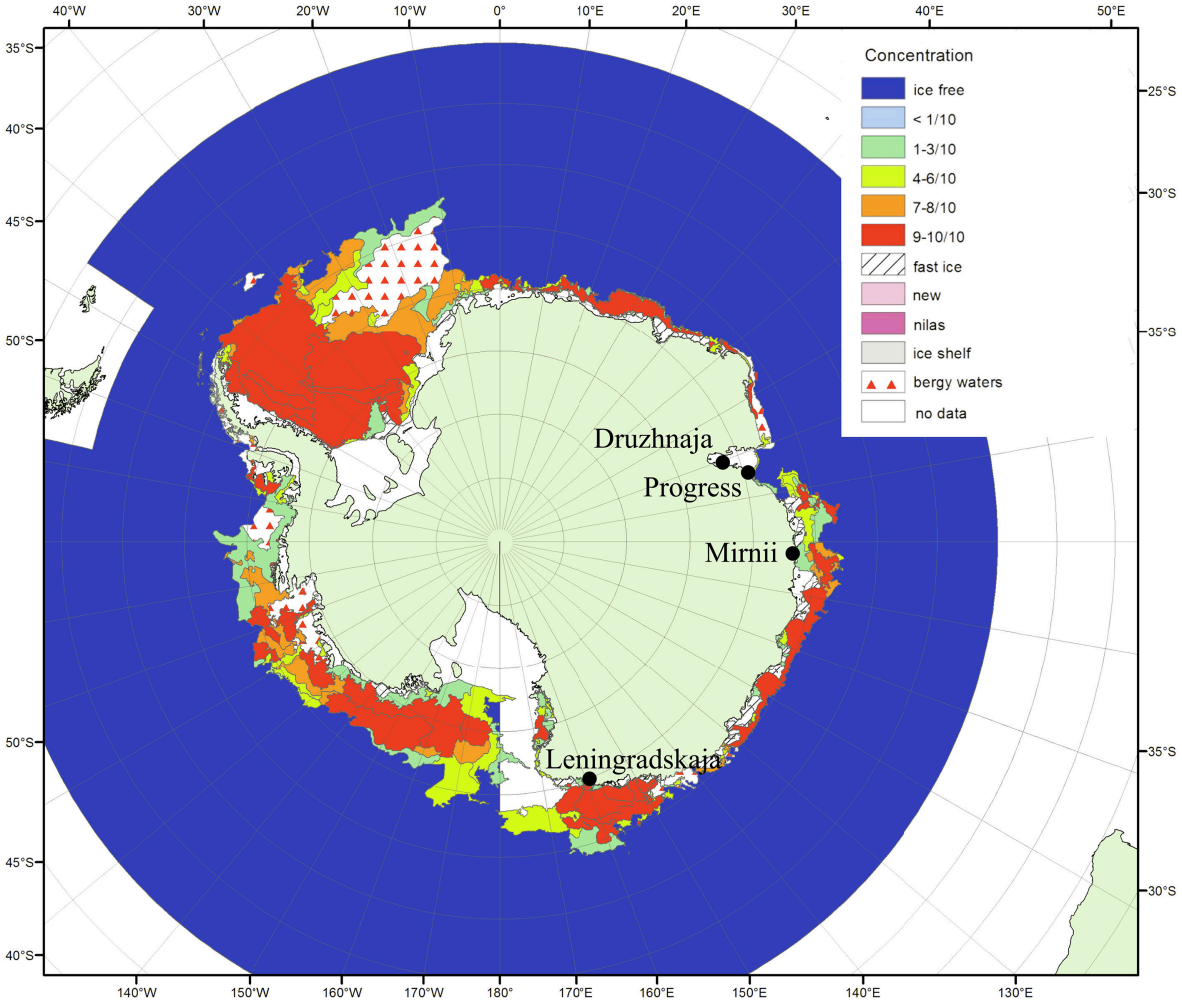
**Antarctic surface snow sampling sites**. The locations of the four Russian research stations where the snow samples were taken are shown on the map of Antarctica (from the archive of Russian Institute of Arctic and Antarctica http://wdc.aari.ru/datasets/d0040/antarc/png/). The color code indicates ice concentration for January 2010 during the time of sampling. The distances from open water for Mirnii and Progress are 1–5 km, for Druzhnaja—150 km, for Leningradskaja—400 km.

Samples were collected during the austral summer of 2009–2010 year from vicinity of four coastal Russian Antarctic stations—Progress, Druzhnaja, Mirnii, and Leningradskaja as described previously (Lopatina et al., 2013). All stations are located on the coastal part of Eastern Antarctica (Figure 1). The distance between stations ranges from ~150 km between Progress and Druzhnaja to ~3000 km between Progress and Leningradskaja. The stations vary in indicators of climatic conditions, such as average temperature, humidity and wind speed as shown in Table 1.

**Table 1:** **Geographical and climatic data for the four sampling sites**.

**Station**	**Geographic coordinates**	**Elevation, m**	**Mean surface air T, °C**	**Mean ground T, °C**	**Mean precipitation, mm**	**Mean surface wind, m/s**
Druzhnaja	69°44′S 72°42′E	No data	No data	No data	No data	No data
Leningradskaja	69°30′S 159°23′E	291	−14.6	−15.4	58.4	8.4
Mirnii	66°33′S 93°01′E	39,9	−11.3	−11.7	43.8	11.3
Progress	69°23′S 76°23′E	14,6	−9.2	−7.4	12.5	5.9

### Total DNA extraction, amplification of 16S rRNA genes, and sequencing

Samples of total DNA were prepared as described previously (Lopatina et al., 2013). PCR of a bacterial 16S rRNA gene fragment (V3-V4 region) was performed with two universal primers 341F (5′-CCTACGGGNGGCWGCAG-3′) and 805R (5′-GACTACHVGGGTATCTAATCC-3′) under general conditions described by Herlemann et al. (2011). 2 ng of total DNA was used as a template for each PCR reaction. To avoid biases during PCR amplification 10 replicates of each PCR reactions were performed for every sample and mixed prior to further manipulations. Amplicons were visualized on 1% ethidium bromide stained agarose gels and purified using Promega Gel extraction kit according to the manufacturer's instructions. Negative controls (an aliquot of 10 l of Milli Q water subjected to concentration and DNA purification for each sample) resulted in no visible amplification products, confirming that our sample collection and processing techniques were essentially free of contamination. Pair-end sequencing was carried out on Illumina MiSeq platform with MiSeq reagent kit v.2 (Illumina, USA) as described previously (Caporaso et al., 2011).

### Sequencing of metagenomic DNA libraries

For metagenomic sequencing 100 ng of total DNA from each sample was used to prepare libraries as described previously (Caporaso et al., 2011). Pair-end sequencing was carried out on Illumina MiSeq platform with MiSeq reagent kit v.2 (Illumina, USA).

### Analysis of 16S rRNA gene and metagemic libraries

Reads produced by sequencing of 16S rRNA amplicons were subjected to basic trimming (Schloss et al., 2011). First, sequences were demultiplexed, trimmed by quality with Phred score ≥ 20 and no admission of ambiguous bases using CLC Genomics 7.0 workbench software (CLC Bio Aarhus, Denmark), and sequences longer than 100 bp were taken for further processing. Homopolymers longer than 8 nt were removed using NGS QC toolkit with HomoPolymerTrimming.pl Perl script (Patel and Jain, 2012) and chimeric sequences were removed using Ribosomal Database Project (RDP) chimera check pipeline (Edgar et al., 2011). Phylotyping and statistical analysis was performed using the RDP classifier via taxonomic supervised method with 80% confidence threshold cut off (Cole et al., 2014), as this approach allows rapid and extensive community comparison (Sul et al., 2011).

Raw reads from shotgun metagenomic sequencing were trimmed by quality with Phred score ≥ 20 and no admission of ambiguous bases. Adapters were trimmed using CLC Genomics workbench software (CLC Bio Aarhus, Denmark); reads longer than 50 bp were subjected to further analysis. Trimmed sequences were applied to MG-RAST database (Meyer et al., 2008). Reads were taxonomically and functionally annotated by similarity searching against M5NR database and Subsystems database, respectively, with default parameters (maximum *e*-value cutoff of 10^−5^, minimum identity cutoff of 60% and minimum alignment length cutoff of 15).

To specifically search for viral sequences in metagenomic libraries, sequences were subjected to Metavir online tool (Roux et al., 2014), where they were blasted against Viral Refseq database (NCBI). Obtained affiliated sequences were filtered from bacterial homologs using supplementary pipeline: firstly, they were blasted against nucleotide (nt) database using blastn standalone application and afterwards viral sequences were extracted using Megan 5.10.1 software (Huson et al., 2011).

### Statistical analysis

Several measurements of alpha diversity were used to estimate the diversity of bacteria in the samples. Species richness estimators S_chao1_ and S_ace_ (Kemp and Aller, 2004b), and community diversity indices Shannon (1948) and Simpson (1949) were calculated using RDP analysis tools. Coverage of 16S rRNA libraries was calculated according to Good's formula: C = 1 – (N/individuals), where N is the number of sequences that occurred only once (Kemp and Aller, 2004a).

### Identification and analysis of CRISPR arrays

To construct a set of CRISPR arrays for each metagenomic dataset we used CRASS algorithm (Skennerton et al., 2013) with default parameters: repeat lengths 23–47 bp, spacer lengths 26–50 bp, and minimum three spacers in array as default parameters. Spacer and repeat sequences were compared with nucleotide (nt) database using BLAST+ tool installed on Galaxy platform with default parameters for short input sequence (word size 7, gapopen 5, gapextend 2, reward 2, penalty -3, *e*-value 0.01). Repeat sequences from identified CRISPR arrays were classified using CRISPRmap tool (Lange et al., 2013). The *cas* genes search was performed using MG-RAST Subsystems annotation tool (Meyer et al., 2008).

To amplify CRISPR arrays of *Flavobacterium psychrophilum* from total DNA samples primers Flavo_F (CAAAATTGTATTTTAGCTTATAATTACCAAC) and Flavo_R (TACAATTTTGAAAGCAATTCACAAC) were used. Amplification reactions were carried out with Taq DNA polymerase under the following conditions: initial denaturation for 5 min at 95°C, followed by 28 cycles of 30 s at 95°C, 30 s at 55°C, and 40 s at 72°C, and a final extension at 72°C for additional 2 min. Amplicons were visualized on 1% ethidium bromide stained agarose gels and DNA fragments of 200–1000 bp in length were purified from the gel and sequenced on Illumina MiSeq platform as described above. Raw reads were demultiplexed, trimmed by quality with Phred score ≥ 20 and no admission of ambiguous bases using CLC Genomics 7.0 workbench software (CLC Bio Aarhus, Denmark).

Spacers from amplified CRISPR arrays were bioinformatically extracted using DNAStringSet function of IRanges package in R. To decrease the amount of spacers and to avoid overrepresented diversity because of mistakes during sequencing, spacers were clustered using a *k*-means algorithm (MacQueen, 1967). The maximum number of substitutions corresponding to biologically similar spacers within one cluster was equal to 5. Coverage and diversity estimates S_chao_ and S_ace_ for total amount of spacers or clusters in each sample were calculated with estimateD function of vegan package in R. Centers of spacer clusters (sequences of mean arithmetic value for each nucleotide position calculated from all spacers within a cluster) were compared against nucleotide collection (nt) and environmental collection (env_nt) databases, as well as against custom-made database containing sequences from Antarctic shotgun metagenomic libraries from the present work, with BLASTn algorithm using default parameters for short input sequences mentioned above and an *e*-value cut off of 0.01. Sequences with < 5 mismatches were considered as positive hits. Metagenomic sequences containing protospacers were blasted against nt and nr databases with default parameters for BLASTn algorithm and an *e*-value cut off of 0.001 using BLAST+ tool installed on Galaxy platform. PAM searches were performed with CRISPRTarget online tool (Biswas et al., 2013). Eight nucleotides upstream and downstream of each protospacer were extracted and used for PAM logo search with Weblogo online tool (http://weblogo.berkeley.edu/logo.cgi).

### Data access

The data of 16S rRNA high throughput sequencing were deposited to MG-RAST database under accession numbers 4616914.3 (Druzhnaja), 4616915.3 (Leningradskaja), 4616916.3 (Mirnii), and 4616917.3 (Progress). The data of shotgun metagenomic sequencing were deposited to MG-RAST database under accession numbers 4624083.3 (Druzhnaja), 4624084.3 (Leningradskaja), 4624085.3 (Mirnii), and 4624086.3 (Progress).

## Results

### Metagenomic analysis of 16S rRNA sequences from Antarctic snow samples

Earlier, we studied the bacterial diversity of surface snow from two Russian Antarctic stations, Leningradskaja and Druzhnaja, by analyzing individual 16S rRNA gene fragments cloned after PCR amplification of DNA from melted snow samples collected during the 54th (2009) and 55th (2010) Russian Antarctic expeditions (Lopatina et al., 2013). For the present work, we used high-throughput sequence analysis of 16S rRNA amplicons from Leningradskaja and Druzhnaja 55th expedition samples analyzed previously and also included samples collected at the Progress and Mirnii stations during the same time. The microbial diversity at the two latter stations was not analyzed before, however, the biological activity of snow collected at Mirnii was at least 10 times higher than in the Leningradskaja and Druzhnaja samples (Lopatina et al., 2013). For Progress, bioactivity levels were low (4.4 pmol/h^*^l of [methyl ^3^H] thymidine incorporation and 33.1 pmol/h^*^l of [^3^H] L-leucine incorporation) and comparable to those in Leningradskaja and Druzhnaja samples.

**Figure 2:**
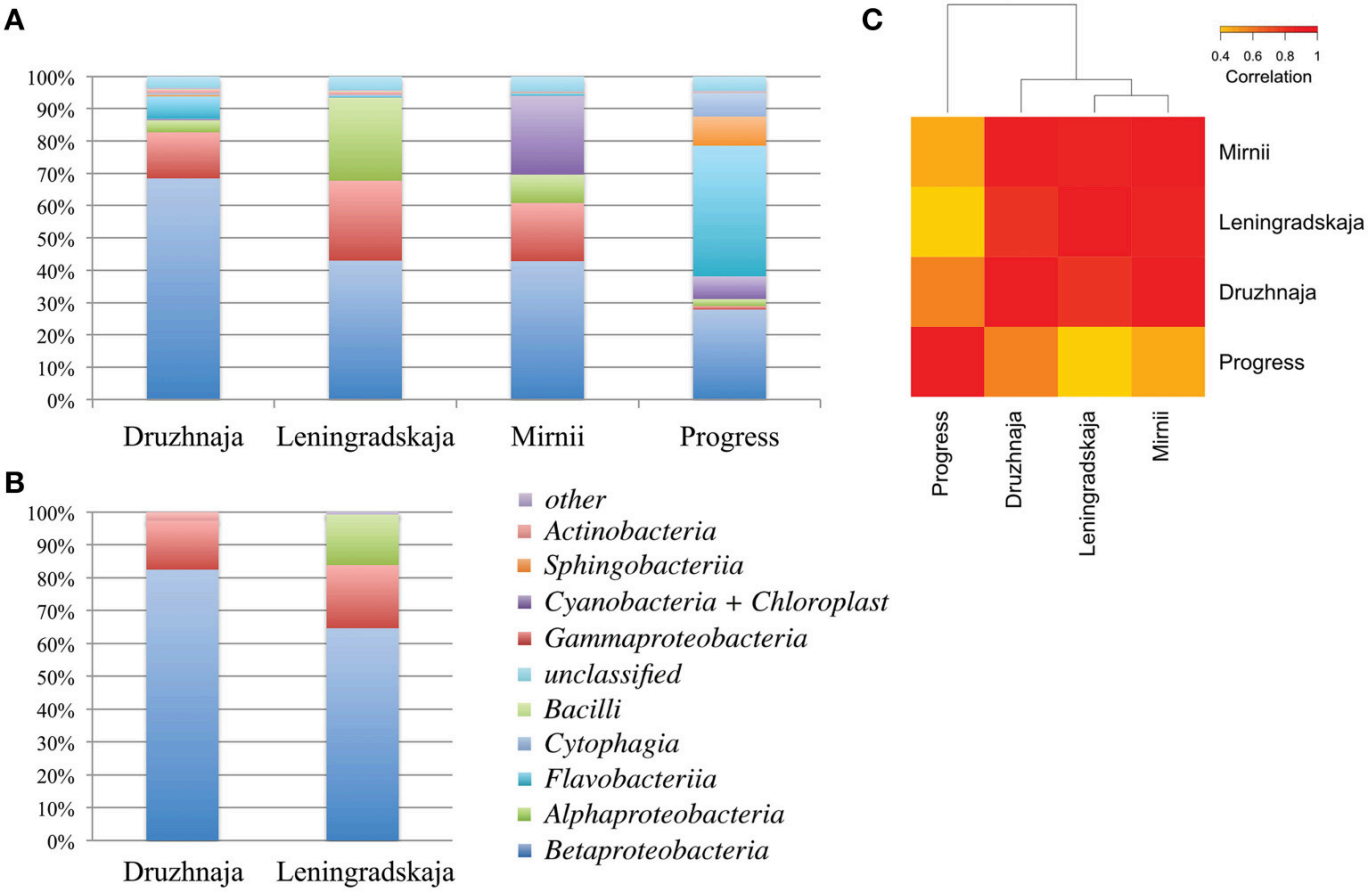
**Class-level bacterial diversity in Antarctic snow samples. (A)** Relative abundance of class-level bacterial taxonomies based on PCR amplifications and high-throughput sequencing of 16S rRNA gene fragments is shown at the top. “Other” group includes minor classes with < 0.6% of total abundance, namely *Verrucomicrobiae, Clostridia, Gemmatimonadetes, Planctomycetia, Deltaproteobacteria, Spartobacteria, Epsilonproteobacteria*, and 14 rare classes found only in one location. **(B)** Similar data using clone library approach for same samples from Druzhnaja and Leningradskaja stations are shown. **(C)** A heatmap comparing class-level bacterial diversity and abundance among the four samples based on high-throughput sequencing results. The colors show the extent of relatedness between the samples as indicated in the legend.

DNA concentration was estimated by measuring absorbance by NanoDrop yielding a concentration estimate of 1, 1, 2, and 14 ng/μl for Druzhnaja, Leningradskaja, Progress and Mirnii samples, correspondently. To access bacterial diversity in snow samples, a fragment of bacterial 16S rRNA gene was amplified from total DNA following by Illumina pair-end high throughput sequencing (HTS). The overall sequencing statistics are presented in Table S1. Results of phylogenetic analysis of 16S rRNA sequences from Leningradskaja and Druzhnaja samples generated by HTS and Sanger sequencing of cloned libraries were first compared. Overall, comparisons of class-level distribution revealed by both methods are in very good agreement with each other (Figure 2B; Pearson coefficient of correlation for Druzhnaja sample–0.99, for Leningradskaja–0.95). Yet, for both stations, HTS analysis revealed increased relative abundance (or even appearance) of several minor classes, including *Flavobacteriia, Alphaproteobacteria, Sphingobacteriia, Cytophaga*, and *Actinobacteria*.

16S rRNA gene sequences recovered by HTS from the four stations fell into 34 classes based on RDP classification. 3.4, 3.9, 4.5, and 4.3% of 16S rRNA gene reads from, correspondingly, Druzhnaja, Leningradskaja, Mirnii, and Progress samples could not be affiliated to any known bacterial class by the RDP classification tool. Overall, the most abundant classes were: *Alphaproteobacteria, Betaproteobacteria, Gammaproteobacteria, Sphingobacteriia, Flavobacteriia, Cytophagia, Actinobacteria, Chloroplast*/*Cyanobacteria, Bacilli*. While *Betaproteobacteria* were dominant in Leningradskaja, Druzhnaja, and Mirnii samples, *Flavobacteriia* were the major class in the Progress sample, constituting 40% of all sequences (Figure 2A). In fact, the latter sample was clearly very different in composition from the first three based on Pearson correlation analysis at class level (Figure 2C).

**Figure 3:**
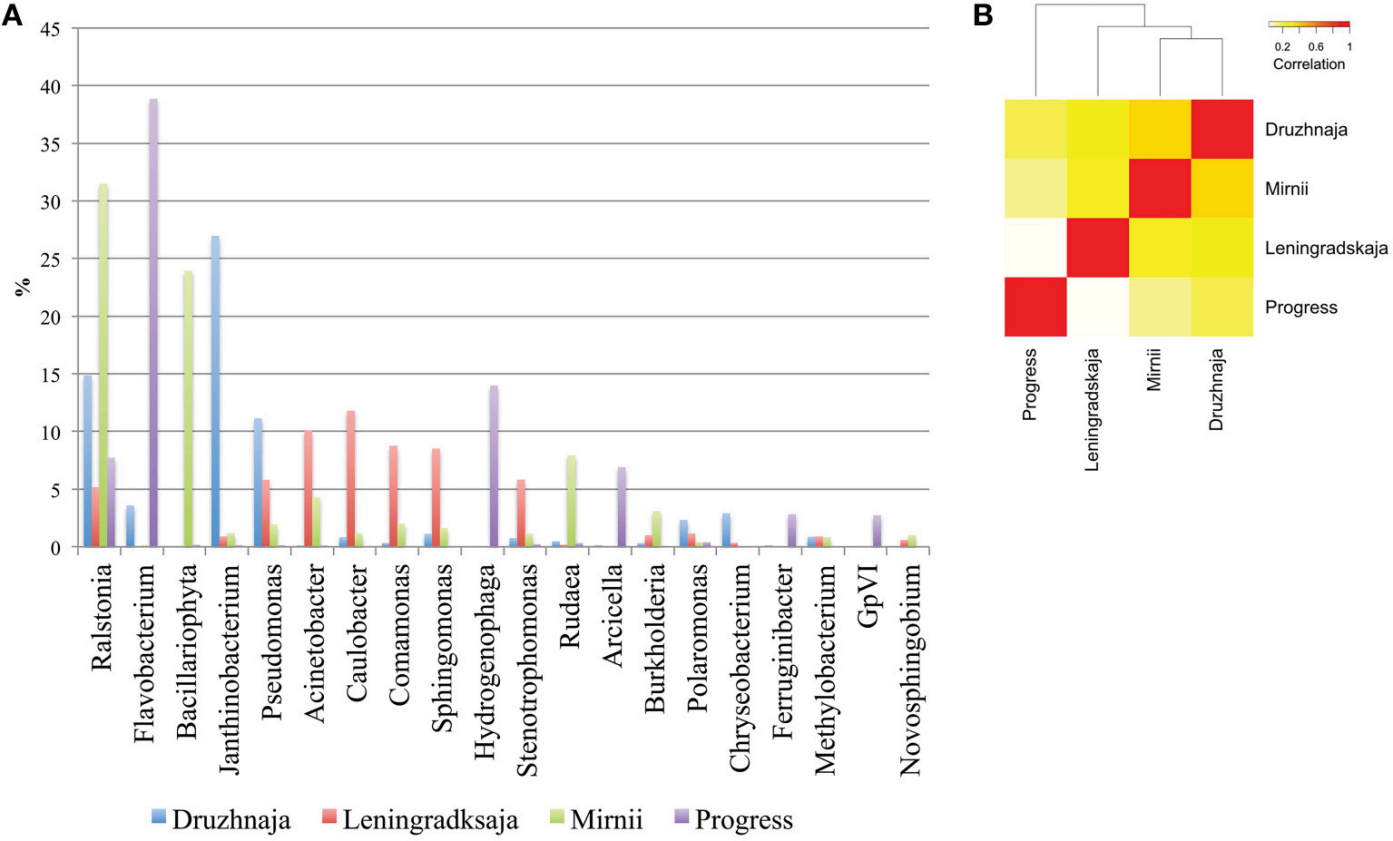
**Genus-level bacterial diversity in Antarctic snow samples based on PCR amplicon library. (A)** Frequencies of reads corresponding to 20 most abundant genera present in all four samples are shown. For each genus, the height of color-coded bars reflects the percentage of corresponding reads in the entire sample from each station. **(B)** A heatmap comparing genus-level bacterial diversity and abundance for 255 genera detected in Antarctic snow samples. The colors show the extent of relatedness between the samples from each station as indicated in the legend.

Deeper taxonomic affiliation analysis at each site was next performed. 28, 20, 14, and 35% of 16S rRNA gene reads from, correspondingly, Druzhnaja, Progress, Mirnii, and Leningradskaja could not be affiliated to any known genus by the RDP tool. Results of the analysis of remaining reads are shown in Figure 3A, where abundances of 20 most prevalent genera are presented. The genus detected in the most abundance in any given sample was *Flavobacterium*, which comprised 39% of the sequences in the Progress library, followed by *Hydrogenophaga* (14%) and *Ralstonia* (7%). In the Druzhnaja sample, 16S rRNA genes from *Janthinobacterium* were dominant (27%), followed by *Ralstonia* (15%), and *Pseudomonas* (11%). In the Leningradskaja sample, 16S rRNA genes from *Caulobacter* (12%), *Acinetobacter* (10%), and *Comamonas* (9%) were most abundant. These genera were also the most abundant during clone library analysis (Lopatina et al., 2013) and in fact the abundance of genera in Druzhnaja and Leningradskaja stations, as revealed by cloning library and HTS approaches, was highly correlated (Pearson correlation coefficient of 0.8 and 0.9, respectively, data not shown). In Mirnii—rRNA gene sequences of *Ralstonia* (31%), *Bacilariophyta* (chloropast-containing diatoms) (24%), and *Rudaea* (8%) were the most dominant. There was no correlation of genera abundance or presence between samples from the four different stations: the Pearson correlation coefficient varied from 0.1 for Progress and Leningradskaja to 0.4 between Mirnii and Druzhnaja (Figure 3B).

### Shotgun metagenomic analysis of antarctic snow DNA samples

DNA samples from the four stations were also subjected to shotgun metagenomic sequencing. The summary of data obtained from four snow samples is shown on Table S2. Sequences that passed the QC criteria were applied to Best hit classification algorithm of the MG-RAST software using M5NR database for phylogenetic affiliation of sequences. The results are summarized in Table 2. The percentage of archaeal sequences in shotgun metagenomic libraries was consistently low in all stations (< 0.2% of all sequences) and these sequences were not further analyzed; no archaeal sequences were obtained previously in clone 16S rRNA libraries in Druzhnaja and Leningradskaja samples (Lopatina et al., 2013). Viral samples were extracted from metagenomic data through Metavirome tool and were also rare. *Eukaryota* were well-represented in Mirnii library—15% of all sequences. Samples from other stations contained much less eukaryotic sequences (~1% or less). More than half of eukaryal sequences from Mirnii were from *Bacilariophyta*, suggesting that “cyanobacterial” sequences present in the amplified 16S rRNA gene samples from this station were actually of chloroplast origin. The Mirnii and Progress stations are located within 1–5 km of open water, while Druzhnaja and Leningradskaja, are, respectively, about 150 and 400 km away (Figure 1). The abundance of *Chloroplasts*/*Cyanobacteria* is thus probably correlated with closeness to open water. Most of metagenomic sequences from all samples corresponded to domain *Bacteria*. Class- and genus-level phylogenetic complexity of bacterial sequences from shotgun and 16S rRNA metagenomic data matched well for all four stations (Pearson coefficient values 0.97–0.99 for class level and 0.68–0.86 for genera level).

**Table 2:** **Overall phylogenetic structure of snow microbial communities**.

**Station**	**Prokaryota, %**	**Eukaryota, %**	**Viruses, %**	**Archaea, %**	**Unclassified, %**
Druzhnaja	98.29	1.41	0.06	0.1	0.15
Leningradskaja	99.04	0.77	0.06	0.06	0.08
Mirnii	84.65	14.97	0.14	0.17	0.20
Progress	98.38	1.22	0.04	0.31	0.32

Protein-coding sequence reads from snow metagenomes were classified to metabolic functions based on Subsystems database using MG-RAST software. The most abundant functional groups were related to housekeeping functions, such as clustering-based subsystems (functional coupling evidence but unknown function; 14-16%), carbohydrate metabolism (9%), amino acid biosynthesis (8%), and protein metabolism (6.5–8.5%). Stress response related genes constituted 2.3–2.9% of all annotated reads and within this group there was a high proportion of oxidative stress genes (43–44%). Genes of photosynthesis and respiration were clearly more abundant at Mirnii station, where chroloplast/cyanobacterial sequences were common.

**Figure 4:**
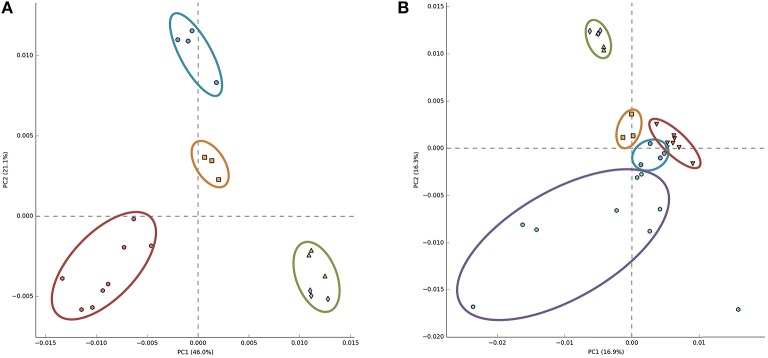
**Principal component analysis of 30 metagenomes from five different environments based on frequencies of COG categories**. A scatter plot of PCA-scores depicting variance of COG categories detected in different environmental metagenomes. In panel **(A)** COGs from Antarctic snow (cyan) are compared to temperate soil samples (red), sea water (green), and Antarctic microbial mat (orange). In panel **(B)** Arctic snow samples (violet) are included in comparison.

Recently, principal component analysis of the relative abundance of annotated reads of functional subsystems from Arctic surface snow metagenomes was presented and a conclusion was made that snow samples grouped together and were well-separated from other ecosystem metagenomes (Maccario et al., 2014). We repeated this analysis including our Antarctic snow metagenomes data. When Antarctic samples were substituted for Arctic samples used in the previous analysis, clear ecosystem clustering similar to the earlier reported result was obtained (Figure 4A), seemingly indicating commonalities of microbial communities of Antarctic snow. However, when Arctic snow metagenomic samples were also included, Antarctic samples became indistinguishable from soil and Antarctic microbial mat metagenomes; the free ocean water samples remained tightly clustered and separate, while the Arctic snow samples became very dispersed (Figure 4B).

### Analysis of CRISPR-Cas sequences in antarctic metagenomes

The CRISPR-Cas systems of adaptive prokaryotic immunity are widespread in bacteria (Marraffini and Sontheimer, 2010; Makarova et al., 2011) and are highly dynamic (Deveau et al., 2008), allowing one, in principle, to monitor the structure of bacterial populations in environment (Bhaya et al., 2011; Sun et al., 2015). We searched for *cas* genes and CRISPR arrays fragments in sequences from our shotgun metagenomic libraries. The *cas* genes of all three CRISPR-Cas system types were found. Specifically, fragments of *cas1, cas2, cas3, csn1* (*cas9*) as well as *cas4b* and *cmr1-6* genes were detected. These reads constituted less than 0.03% of all sequences. Fragments of CRISPR arrays were also identified in every library. Some identified repeats matched previously described ones, for example a 46-bp repeat from type II CRISPR-Cas system from *Flavobacterium psychrophilum* (Touchon et al., 2011), found in Progress and Druzhnaja, and a different type II 36-bp repeat matching *Flavobacterium columnare* in Leningradskaja and Progress samples. A type I-F CRISPR-Cas system repeats from *Yersinia pseudotuberculosis* were found in Druzhnaja, Leningradskaja, and Progress (Table S3). CRISPRmap, an automated tool for classification of prokaryotic repeats based on sequence and structure conservation, has been reported to classify as many as 30–40% of repeat sequences from human microbiome samples (Lange et al., 2013). In contrast, in the case of Antarctic samples out of a total of 40 distinct repeats identified, only one could be matched with a known family (six could be matched with a known structural motif), indicating that the variety of existing adaptive immunity systems is greatly underexplored.

When spacers extracted from identified Antarctic CRISPR arrays were analyzed, no matches with spacers of previously known CRISPR arrays was detected. Further, when the entire collection of 570 unique spacers recovered from Antarctic snow metagenomic libraries was analyzed against the NCBI nucleotide collection (nt), only a single hit, for a spacer associated with the *F. columnare*-like 36-bp repeat, was found. This spacer matched exactly a fragment of 16S rDNA sequence of another representative of the *Flavobacterium* genus, *Flavobacterium* sp. 136G (NCBI accession number KM021132.1), contrary to the general observation that CRISPR spacers target DNA of mobile genetic elements.

**Figure 5:**
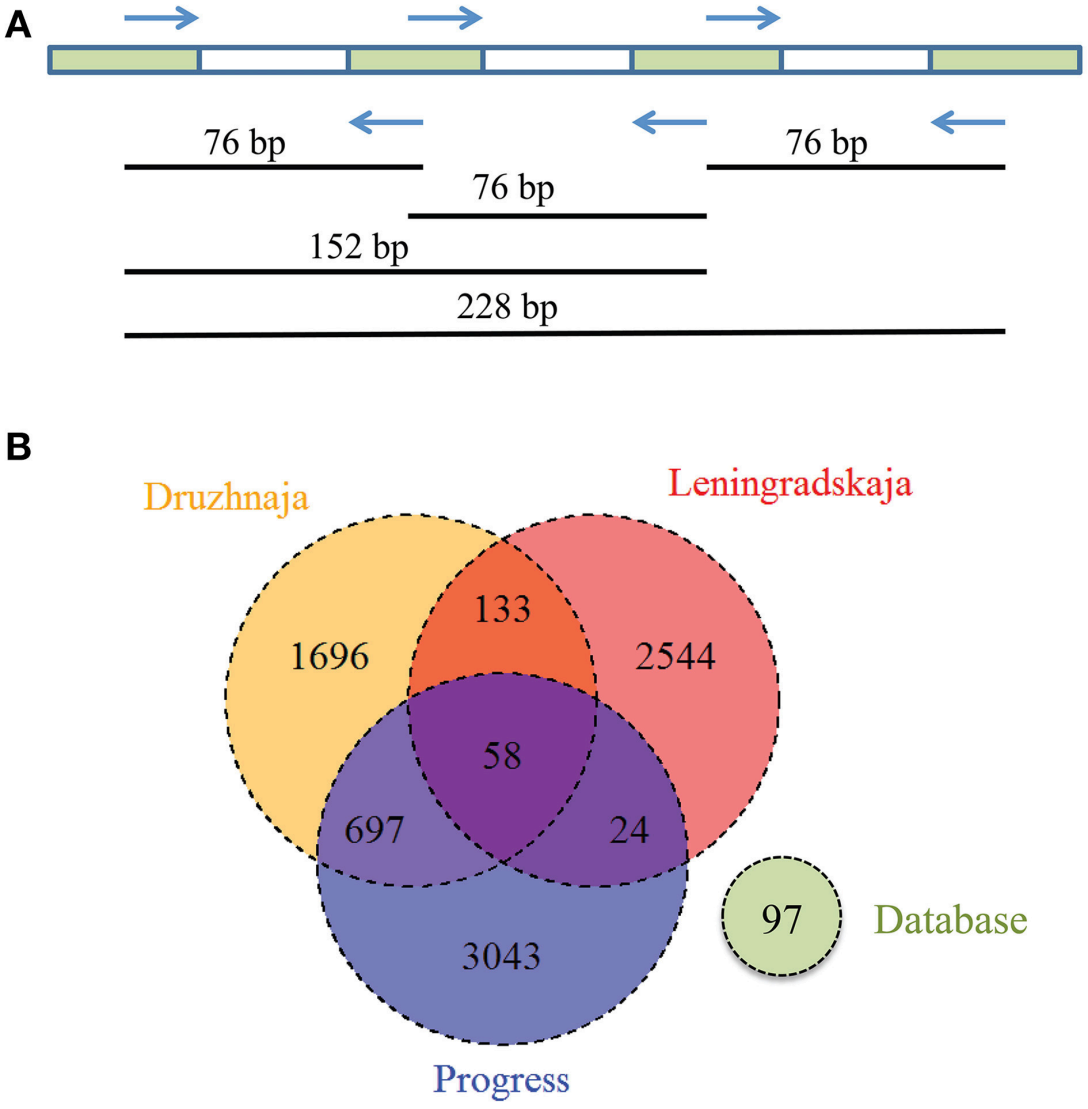
**Analysis of ***Flavobacterium physchrophilum*** CRISPR spacers in Antarctic snow samples. (A)** A strategy used to amplify spacers associated with *F. physchrophilum* CRISPR repeats from environmental samples, the length of amplified fragment corresponds to the number of containing spacers. **(B)** A Venn diagram showing the number of shared and unique clusters of spacers associated with *F. physchrophilum* CRISPR repeats in three Antarctic snow samples. Known *F. physchrophilum* spacer clusters from 10 publically available genomes are also shown (“database”).

CRISPR interference in type II systems requires a functional protospacer adjacent motif (PAM), located downstream of the protospacer (Chylinski et al., 2014). The PAM sequence of *F. columnare* type II CRISPR-Cas system is not known. Analysis of 43 spacers from CRISPR array of a sequenced *F. columnare* genome (NCBI accession number CP003222.2) revealed four matches with flavobacterial phage FCL-2 protospacers. Sequences adjacent to these protospacers contained a TAA trinucleotide five nucleotides downstream of each protospacer. Both the downstream location of the putative PAM, and its separation from protospacers by a string of non-conserved nucleotides is typical for type II CRISPR-Cas systems (Chylinski et al., 2014). The putative PAM sequence was absent downstream of the *Flavobacterium* sp. 136G 16S rDNA sequence matching the spacer identified from metagenomic data. Thus, the particular 16S rDNA targeting spacer may not be functional (see, however, below). Three spacers—associated with the *F. psychrophilum* 46-bp repeat—were found in both Progress and Druzhnaja samples. The rest of the spacers were unique for each station. Since flavobacterial rRNA was present in samples from all spacers, we were interested in assessing diversity of *F. psychrophilum* spacers in each site. To this end, PCR primers matching 46-bp repeat were designed and used to amplify spacers from each snow community DNA (Figure 5A). By design, the procedure allows amplification of spacers associated with the 46-bp repeat, however the information about the order of the spacers in CRISPR arrays is lost. Amplification products were detected in samples from three stations—Progress, Druzhnaja, and Leningradskaja. The amplified material was subjected to Illumina sequencing. A total of ~870,000 spacers with an average length of 30 ± 2 nucleotides was obtained (in published *F. psychrophilum* genomes spacers are 28–31 long). We next clustered spacers in each sample (MacQueen, 1967), combining spacers that differ from each other by < 5 nucleotides in the same cluster. After clustering, 2759 unique spacer clusters remained in Leningradskaja, 2584—in Druzhnaja, and 3822—in Progress station (Table 3, Supplementary Dataset S4). The calculated coverage of the three cluster libraries ranged from 40% for Druzhnaja to 61% for Progress samples (Table 3), so true variety in samples was thus 1.5–2.5 times higher than the actual number of clusters obtained. It therefore follows that the diversity of CRISPR spacers associated with the *F. psychrophilum* 46-bp repeat (and, by extension, of *F. psychrophilum*) in Antarctic snow is extremely high. When spacers from each station were compared to each other, only 58 clusters (0.7% of the total) were common for all three stations (Figure 5B). The percentage of clusters unique to each station varied from 66% for Druzhnaja to 92% in Leningradskaja. The Druzhnaja spacer set was most similar to Progress (about 30% of common spacers), with much smaller (< 7%) overlap with Leningradskaja set. The overlap of Progress and Leningradskaja sets was just 3%. Ninety-five percent of all spacers were located within 14, 29, and 21% of clusters from Progress, Leningradskaja, and Druzhnaja, correspondently, i.e., were highly overrepresented. Bacteria with such spacers must be highly abundant in the samples. Alternatively, overrepresented spacers may be shared between many strains.

**Table 3:** **Statistics of high-throughput sequencing of PCR amplified Antarctic ***Flavobacterium psychrophilum*** CRISPR spacers and spacer clustering results**.

**Station**	**# of reads**	**# of spacers**	**Clusters**					
			**# of clusters**	**% of unique clusters**	**C_chao1_, %**	**C_ace_, %**	**S_chao1_**	**S_ace_**
Druzhnaja	284,286	273,255	2584	65.6	48	40	6382 ± 386	5359 ± 42
Leningradskaja	321,550	313,241	2759	92.2	43	47	5824 ± 303	6477 ± 48
Progress	263,548	255,447	3822	79.6	60	61	6271 ± 170	6332 ± 46

A small fraction (1–3%) of self-complementary spacers derived from the same protospacer was observed. Such pair-mated spacers have been reported before for *Streptococcus agalactiae, Sulfolobus solfataricus*, and *Escherichia coli* (Erdmann and Garrett, 2012; Lopez-Sanchez et al., 2012; Shmakov et al., 2014). In most cases, when self-complementary spacers were observed, one spacer in the pair belonged to an over-represented group. A high number of such paired spacers were shared between two or more stations (up to 92% self-complementary spacers in the Druzhnaja station sample were also found in other stations).

Many reads corresponded to amplified fragments that contained two spacers and, therefore, harbored a copy of an “internal” repeat, whose sequence, by design, could not be affected by the primers used during amplification step (Figure 5A). Analysis of such reads revealed different repeat variants (Table S5). Similar cases of nearly identical repeats sequences were described previously for other organisms, for example, *E. coli* (Touchon and Rocha, 2010) or *H. volcanii* (Maier et al., 2013). The most abundant variant constituted 65.6% of all “internal” repeat sequences and matched the published *F. psychrophilum* repeat consensus used to design oligonucleotide primers for amplification. The second variant had one mismatch from consensus in the 6th position and constituted 34% of all “internal” repeats. Two other repeat variants had, in addition to the 6th position consensus mismatch, changes in the 13th or the 21th positions and were minor (0.2 and 0.1% of all “internal” repeats, correspondingly). The relative proportion of repeat variants was the same in libraries from the three Antarctic sites analyzed. In sequenced *F. psychrophilum* genomes a variant repeat with one mismatch from consensus in the 18th position constitutes 4% of all repeat sequences. This variant is absent from Antarctic samples.

**Figure 6:**
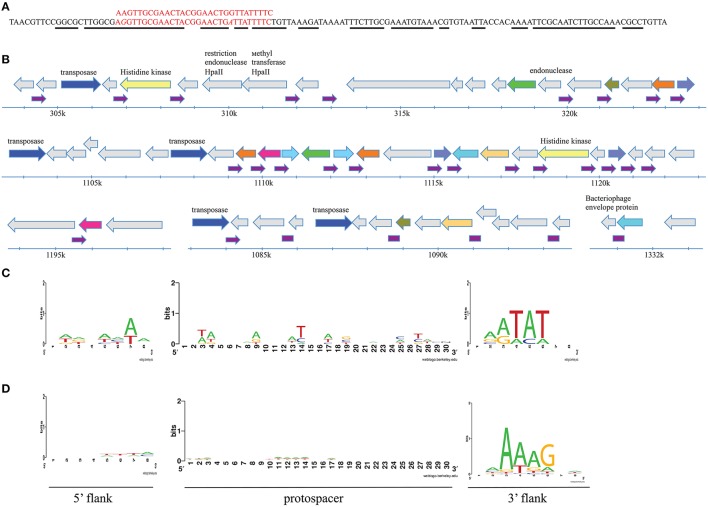
**Analysis of sequences matching spacers associated with ***F. psychrophilum*** CRISPR repeat. (A)** The alignment of sequences of cluster consensus and 125 bp-long repeat. The aligned nucleotide positions are shown in red. Nucleotide positions in 125 bp-long repeat that participate in secondary structure formation of the transcript are underlined with a black line. **(B)** At the top, the sequence of a non-coding 125 bp-long imperfect palindromic repeat and matching *F. psychrophilum* spacer are shown. The distribution and genetic neighborhoods of 30 125 bp-long repeats in regions of *Flavobacterium indicum* genome is shown below. The repeats are indicated by small purple arrows. Genes are shown as arrows. Unlabeled gray arrows correspond to unique open reading frames with unknown function. Unique open reading frames with predicted functions are also shown in gray with annotations. Arrows marked with the same (non-gray) color correspond to genes encoding homologous proteins (more than 80% identity) of unknown function. Genes coding for IS110 family transposes are shown in blue. **(C)** A LOGO showing the results of alignment of 12 flavophage protospacers and their 8-bp flanking regions matching CRISPR spacers from *F. psychrophilum* genomes deposited in the GenBank. **(D)** As in **(C)** but showing the results of alignment of 511 sequences from Antarctic metagenomic matching Antarctic *F. psychrophilum* CRISPR spacers.

When cluster consensus sequences from each station were compared to the NCBI nucleotide database using BLASTn algorithm a very large number of matches with likely irrelevant (i.e., eukaryotic) sequences was found. We therefore limited comparisons to a custom database containing all known sequences of *Flavobacterium* and their phages. None of Antarctic spacers matched any of the 117 unique spacers associated with 46-bp repeat from fourteen sequenced *F. psychrophilum* strains available in the Genbank (our clustering procedure combined these 117 spacers into 97 clusters). Ten Antarctic spacer clusters matched flavobacterial phages FCL-2, 6H, 11b, or 1/32, while 38 matched *Flavobacterium* chromosomes (Table S6). Interestingly, one cluster consensus sequence (leningradskaja_747) had multiple hits in various flavobacterial genomes (*F. indicum, F. psychrophilum, F. columnare*, and *F. branchiophilum*). Inspection of genomic sites that matched this spacer revealed that they are composed of non-coding 125 bp-long imperfect palindromic repeats that are spread throughout the *F. indicum* (30 copies) and *F. psychrophilum* (5 copies) genomes and are present in single copies in *F. columnare* and *F. branchiophilum* (Figure 6A). Analysis of distribution and genetic neighborhoods of these repeats in *F. indicum* and *F. psychrophilum* (data not shown) genomes revealed that they are clustered in regions containing multiple repeated genes of unknown function, transposes genes, and restriction-modification system genes (Figure 6B).

We also analyzed CRISPR cassettes from all *F. psychrophilum* isolates available in the Genbank. Twelve spacers matching flavobacterial phages 6H and 1/32 were identified among the 117 unique spacers present in *F. psychrophilum* strains sequenced to date. When flanking sequences of these protospacers were compared to each other, a likely PAM, NNATAT, downstream of protospacers was detected (Figure 6C). Neither 10 protospacers in the genomes of flavophages nor 38 protospacers in flavobacterial genomes matching Antarctic spacers contain such (or any other) adjacent conserved motive.

We next compared consensus sequences of Antarctic spacer clusters with metagenomic reads obtained in this work as well as with sequences from the metagenomic env_nt database. A total of 117 hits to env_nt database and 511 hits to Antarctic reads was obtained. When the origin of 511 Antarctic metagenomic reads that contained sequences matching *F. psychrophilum* spacers was investigated, 62% of reads could not be identified by either nt or nr database searches. Of the remaining 38% of reads (corresponding to 194 cluster consensus sequences), 87 originated from flavobacterial chromosomes, 21—from *Flavobacterium* phage 11b or plasmids, 49–from other phages (mostly *Cellulophaga* phage phi10:1), and 37 originated from other eubacterial genomes. 12 and 18 additional hits to *Flavobacterium* chromosomes and flavophages, correspondingly, were obtained when reads with no matches to nt database were analyzed against the nr database. Among matching sequences in the env_nt database, there were four *Flavobacterium* chromosomes and 12 bacteriophages of various hosts. When flanking sequences of protospacers identified in Antarctic metagenomic sequences were compared to each other, an area of strong conservation 3-6 nucleotides downstream of the protospacer—NNAAAG - was detected (Figure 6D). This sequence is different from the putative PAM motif detected during searches with spacers from published *F. psychrophilum* genomes (NNATAT, above, Figure 6C) but the location of conserved positions is the same. No conservation in flanking sequences was detected for protospacers identified in metagenomic reads from the env_nt database. Neither one of the putative PAM motives is associated with protospacers from 125 bp-long imperfect palindromic repeats (above).

## Discussion

In this work, we significantly extended the previous analysis of surface snow microbiota around Russian research stations in Eastern Antarctica by (i) increasing the number of stations analyzed, (ii) using high-throughput sequencing to analyze 16S rRNA genes; (iii) performing metagenomic analysis of snow microbiome, and (iv) analyzing the diversity of CRISPR spacers of flavobacteria common in Antarctic snow. Analysis presented in this work was more extensive than previous limited analysis using cloned 16S rRNA genes fragments (~50,000 sequences per each sample compared to ~120 sequences analyzed using clone library approach). Yet, for the two stations where direct comparisons are possible, Druzhnaja and Leningradskaja, a very good correlation between the class- and genus-level composition of microbial sequences in the samples was revealed, indicating that limited sampling of clone libraries did not introduce significant biases in representation of major classes and genera. Moreover, when rRNA gene sequences were extracted from metagenomic reads and class-level phylogenetic complexity was compared with amplified 16S rRNA genes a good match was also observed (Pearson coefficient values between 0.94 and 0.98), indicating that our conditions of PCR amplification of 16S rRNA gene fragments did not introduce significant biases. HTS analysis revealed increased abundance (or even appearance) of several minor classes, including *Flavobacteriia, Alphaproteobacteria, Sphingobacteriia, Cytophaga*, and *Actinobacteria* in both stations These minor classes appeared at the expense of *Betaproteobacteria*, which, nevertheless still remained the major class in both samples. The result is an expected consequence of much deeper coverage obtained with HTS.

Principal component analysis of the relative abundance of annotated reads of functional subsystems from Antarctic surface snow metagenomes revealed some clustering, which, however, was found to be very sensitive to the inclusion of additional environmental samples in the analysis. As expected and recently confirmed by experimental data (Hultman et al., 2015), there is a much greater overlap in shared genes revealed by metagenomic DNA analysis compared to transcriptomic and proteomic analyses of samples from different ecosystems. Such a large overlap may explain the observed instability of results of principal component analysis of functional subsystems in Antarctic metagenomic data. Additional studies will be needed to confirm if there is a characteristic set of gene functions in snow communities.

Spoligotyping, a procedure based on comparisons of spacer sets in different strains of same bacterial species is commonly used for epidemiological tracing of pathogens (Gori et al., 2005). We reasoned that *F. psychrophilum* CRISPR arrays, if present in all four sampled Antarctic sites, may allow us to compare diversity of resident *F. psychrophilum* populations and establish relationships between them. An efficient procedure was elaborated to amplify spacer sets from environmental DNA and k-mean clustering allowed us to parcel the very large number of spacers generated after PCR amplification into a manageable number of spacer clusters. Still, a very high number of spacer clusters was observed in the samples, which is an unexpected result, since a recent report indicated that the *F. psychrophilum* CRISPR-Cas system is inactive and that the spacer content of CRISPR arrays is identical in *F. psychrophilum* isolated in geographically remote locations at different times (Castillo et al., 2015). Spacer sets present in three different Antarctic sites, where successful amplification using *F. psychrophilum* CRISPR repeat-specific primers was achieved differed significantly from each other, with only a very minor portion of spacers being common to all three sites. The larger amount of common spacers between Druzhnaja and Progress agrees with geographical proximity of these stations. Curiously, this similarity, based on common CRISPR spacers was not supported by phylogenetic analysis of bacterial communities based on 16S rRNA genes, according to which Druzhnaja was more similar to Leningradskaja station.

Despite the very large number of *F. psychrophilum* spacers uncovered in our work, no matches with spacers present in *F. psychrophilum* isolates from the Northern hemisphere available in Genbank were observed. Moreover, comparisons with environmental metagenomic data revealed that Antarctic shotgun metagenome from our work, which is orders of magnitude smaller than combined metagenomes stored in the env_nt database contains several times more hits with Antarctic *F. psychrophilum* spacers revealed during HTS analysis of amplified CRISPR spacers. The result suggests that Antarctic *F. psychrophilum* tend to acquire spacers locally. Recent evidence of genetically different pools of viruses in Southern Ocean and Northern hemisphere sampling sites (including Vancouver Island in British Columbia, Monterey Bay, California, and Scripps Pier in San Diego, California) was recently obtained (Brum et al., 2015). The presence of such separate pools in flavophages could be responsible for observed variations in spacer content (see, however, below). The CRISPR-Cas systems of Antarctic *F. psychrophilum* and strains isolated in the Northern hemisphere may even have evolved different PAM specificities since putative PAMs revealed by comparisons of protospacers matching spacers known for the two sites result in different PAMs. Such a result is not without precedent since varying preferences for PAM selection during spacer acquisition were previously noted for type I-E CRISPR-Cas system variants from different *E. coli* strain (Westra et al., 2012) and for type I-B CRISPR-Cas system of *Haloferax volcanii* (Fischer et al., 2012). The presence of different, non-overlapping sets of CRISPR repeat polymorphisms in our Antarctic samples and in known *F. psychrophilum* CRISPR arrays also supports existence of local variations.

The original theoretical insights about the immune function of CRISPR-Cas systems came after observation of matches between spacer sequences and protospacers in bacteriophage and plasmid sequences specific to a bacterial host (Makarova et al., 2006). Later, self-targeting spacers were also identified and a regulatory function of such spacers was proposed (for detailed review, see Westra et al., 2014). Analysis of *F. psychrophilum* repeat associated spacers suggests, that at least for the Antarctic spacer set, targeting of bacteria related to the host is the most common scenario. Such targeting could help prevent genetic exchange between the species within the genus, although the biological significance of such restriction is unclear.

Previous analysis has revealed the loss of synteny within the *Flavobacterium* spp. genomes likely due to the presence of numerous repeats (e.g., insertion sequences and the *rhs* elements (McBride et al., 2009; Touchon et al., 2011). Our analysis revealed an interesting case of a CRISPR spacer with multiple hits in various flavobacterial genomes. The matching sequence was part of a non-coding 125 bp-long imperfect palindromic repeat that is spread throughout the *F. indicum* and *F. psychrophilum* genomes and is also present in single copies in *F. columnare* and *F. branchiophilum*. The location and the number of these repeats differ in different isolates of *F. psychrophilum*, suggesting that they are subject to horizontal transfer. The 125-bp repeat is distinct from either IS or *rhs* elements, however, it may play a similar role in promoting flavobacterial genome plasticity. Targeting of this element by the CRISPR-Cas system may help control the spread of such elements and is in line with an emerging theme that CRISPR-Cas systems serves as one of the mechanisms of endogenous gene regulation (Westra et al., 2014).

Our analysis of Antarctic spacers has an important caveat in that we determine the identity of spacers associated with a particular repeat and can not exclude that such a repeat (and spacers) are not coming from arrays from other, non-*F. psychrophilum* arrays. We consider this scenario unlikely since at least in Progress station, where rRNA gene sequences from *F. psychrophilum* are most abundant, the spacer variety is also the largest. Besides, the largest number of spacers with matches to metagenomic sequences match *Flavobacterium* chromosomes, which also strengthens the link between spacers identified by our approach and the *Flavobacterium* genus.

## Author contributions

AL collected samples, performed experiments, analyzed data, prepared figures; SM performed clustering and PCA analysis, prepared figures; SS performed heatmap analysis; ML performed NGS sequencing; VK collected samples, organized expedition fieldwork; KS designed research, supervised the project, analyzed data, wrote the paper.

## Funding

This work was supported by foundation of Ministry of education and science of the Russian Federation (No14.B25.31.0004) and by Russian Science Foundation (No14-14-00988). The funders had no role in study design, data collection and interpretation, or the decision to submit the work for publication.

### Conflict of interest statement

The authors declare that the research was conducted in the absence of any commercial or financial relationships that could be construed as a potential conflict of interest.
