# Supplementary material for: Metagenomic Analysis of Bacterial Communities of Antarctic Surface Snow
Source: Front Microbiol. 2016 Mar 31;7:398. doi: 10.3389/fmicb.2016.00398 (PMC4814470; doi:10.3389/fmicb.2016.00398)
Supplement: Supplementary file 1 [file Table1.PDF]

Table S1. MG-RAST IDs, raw reads statistics, and diversity metrics of 16S rRNA Illumina reads

Stations	MG-RAST ID	# of sequences after quality trimming	# of genus observed	Chao1 index	Cace index	Shannon index	Simpson index	Coverage, %	# of sequences in cloned library	# of genus observed in cloned library
Druzhnaja	4616914.3	39,483	179	213	212	2.66	0.87	93	117	11
Leningradskaja	4616915.3	61,231	200	231	236	2.82	0.90	93	126	16
Mirnii	4616916.3	50,135	181	224	219	2.57	0.83	90	ND	ND
Progress	4616917.3	46,261	194	234	250	2.51	0.81	76	ND	ND
