# Supplementary material for: Metagenomic Analysis of Bacterial Communities of Antarctic Surface Snow
Source: Front Microbiol. 2016 Mar 31;7:398. doi: 10.3389/fmicb.2016.00398 (PMC4814470; doi:10.3389/fmicb.2016.00398)
Supplement: Supplementary file 2 [file Table2.PDF]

Table S2. MG-RAST IDs, raw reads statistics of shotgun metagenomic sequences

Stations	MG-RAST ID	# of sequences	Totally, bp	# of rRNA genes	# of predicted proteins with known functions	# of predicted proteins with unknown function	no rRNA genes or predicted proteins
Druzhnaja	4624083.3	101,717	29,907,754	799	71,125	20,320	0
Leningradskaja	4624084.3	315,145	70,173,778	1,713	229,753	64,599	0
Mirnii	4624085.3	273,540	76,130,151	1,862	88,170	154,242	10,553
Progress	4624086.3	104,834	31,256,950	331	64,187	31,495	105
