# Supplementary material for: Metagenomic Analysis of Bacterial Communities of Antarctic Surface Snow
Source: Front Microbiol. 2016 Mar 31;7:398. doi: 10.3389/fmicb.2016.00398 (PMC4814470; doi:10.3389/fmicb.2016.00398)
Supplement: Supplementary file 4 [file Table5.PDF]

Table S5. Antarctic *Flavobacterium psychrophilum* CRISPR repeat types

Variants of sequences of flavobacterial repeats	# of reads with particular repeat sequence		
	Druzhnaja	Leningradskaja	Progress
GTTGGTAATTATAAGCTAAAATACAATTTTGAAAGCAATTCACAAC	149,900	172,353	152,926
GTTGGGAATTATAAGCTAAAATACAATTTTGAAAGCAATTCACAAC	87,968	88,890	75,732
GTTGGGAATTATAAGCTAAACTACAATTTTGAAAGCAATTCACAAC	666	1,130	484
GTTGGGAATTATGAGCTAAAATACAATTTTGAAAGCAATTCACAAC	357	223	270
